# Interplay between Tumor Microenvironment and Cancer Cells

**DOI:** 10.1155/2016/4650498

**Published:** 2016-07-12

**Authors:** Kallesh D. Jayappa, Ramesh C. Kovi, Sumanta Chatterjee

**Affiliations:** ^1^Department of Microbiology, Immunology, and Cancer Biology, University of Virginia, Charlottesville, VA 22908, USA; ^2^Experimental Pathology Laboratories Inc. and National Toxicology Program, NIEHS, Research Triangle Park, NC 27709, USA; ^3^Department of Immunology and Regenerative Medicine Program, University of Manitoba, Winnipeg, MB, Canada R3E 0W2

Cancer cell interaction with microenvironment plays a very important role in cancer cell survival, proliferation, and/or metastasis. The cancer cell-microenvironment interaction is a highly complex process and has been one of the leading areas of research in cancer biology. Over the last decade, several components in tumor microenvironment have been identified to have a role in cancer cell survival, proliferation, or metastasis. The underlying mechanisms of tumor cell-microenvironment interactions have been investigated and exploited for cancer therapy. The present issue covers several aspects of cancer cell and tumor microenvironment. This issue contains interesting articles, which were accepted after a rigorous peer review process.

The monocarboxylate transporters (MCTs) are proton-linked plasma membrane transporters that mediate transport of monocarboxylates such as pyruvate and lactate across cellular membranes. Tumor cells depend heavily on anaerobic glycolysis, which generates enormous amount of lactic acid as a byproduct. Lactic acid is toxic to cells and has to be excreted out of the cells through MCTs. An interesting meta-analysis paper by C. D. Bovenzi et al. showed the high expression of MCT4 and CD147 in multiple cancer cells. The elevated MCT4 and CD147 levels were strongly associated with poor overall survival of cancer patients. This work highlighted the importance of MCT4 and CD147 expression in several different cancers and establishes rationale for targeting these molecules for cancer therapy.

As discussed above, as well as by several other reports, mostly the overabundance of ion channels in biological membranes has been implicated in pathogenesis of cancers. Only recently did few studies identify antitumor function of ion channels in some type of cancers. Interestingly, the paper by Z. Xia et al. showed the overexpression of potassium ions in liver cancer cells by the treatment with potassium ions that trigger cancer cell apoptosis, providing convincing evidence in favor of tumor suppression function of potassium ion channels in liver cancer. Given this, the levels of potassium ions in tumor microenvironment can influence the survival of liver cancer cells. Further investigation in this regard is warranted.

Lymphatic vessels are integral part of tumor microenvironment. Although lymphatic vessels were mostly viewed as passive transporters of cancer cells, recent studies demonstrated the active role of these vessels in cancer metastasis. Indeed, these studies collectively established the rationale for targeting lymphangiogenesis for cancer treatment. The paper by X.-L. Ding et al. describes the antilymphangiogenesis effects of Gekko Sulfated Glycopeptide (GSPP) and discusses the potential therapeutic benefits of GSPP in colon carcinoma. The future studies could explore the translational potential of GSPP or related molecules in colon carcinoma or related cancers.

The hormones play a key role in several types of cancers. Gender differences in hormone can influence several aspects of cancer. The paper by S. Caceres et al. developed a male mice xenograft model for mammary tumor and studied the impact of gender differences in hormones on mammary tumor cell proliferation and metastasis. Their preliminary analysis showed the less aggressive nature of mammary tumor cells in male mice compared to female. This work elaborates on the vast literature that describes the importance of extracellular hormone in tumor cell behavior.

The traditional Chinese medicines are being used for cancer treatment for centuries. The review article by J. Xu et al. provides a very nice overview of the selected traditional Chinese medicines that suppress cancer cells by interfering with tumor microenvironment. This article also discusses the synergistic benefits of combining few selected traditional Chinese medicines.

Collectively, this issue will advance our researchers and readers knowledge on various aspects of tumor cell-microenvironment interactions and will provide insight into new research focused in this area and potential therapeutic interventions.

*Kallesh D. Jayappa* *Ramesh C. Kovi* *Sumanta Chatterjee*

